# Establishment and characterization of a penile cancer cell line, penl1, with a deleterious TP53 mutation as a paradigm of HPV-negative penile carcinogenesis

**DOI:** 10.18632/oncotarget.10098

**Published:** 2016-06-16

**Authors:** Jieping Chen, Kai Yao, Zaishang Li, Chuangzhong Deng, Liangjiao Wang, Xingsu Yu, Peili Liang, Qiankun Xie, Peng Chen, Zike Qin, Yunlin Ye, Zhuowei Liu, Fangjian Zhou, Zhenfeng Zhang, Hui Han

**Affiliations:** ^1^ Sun Yat-sen University Cancer Center, State Key Laboratory of Oncology in South China, Collaborative Innovation Center for Cancer Medicine, Guangzhou, China; ^2^ Department of Urology, Sun Yat-sen University Cancer Center, Guangzhou, China; ^3^ Department of Endocrinology, The First Affiliated Hospital of Sun Yat-sen University, Guangzhou, China; ^4^ Center of Medical Imaging & Image-Guided Therapy, Sun Yat-sen University Cancer Center, Guangzhou, China; ^5^ Department of Gynecology, Sun Yat-sen Memorial Hospital, Guangzhou, China; ^6^ Department of Urology, Affiliated Tumor Hospital of Xinjiang Medical University, Urumchi, China

**Keywords:** penile squamous cell carcinoma, lymph node metastasis, characterization, TP53, human papilloma virus-induced penile carcinogenesis

## Abstract

**Purpose:**

To establish penile cancer (PeCa) cell lines for the study of molecular mechanisms of carcinogenesis and testing therapeutic reagents.

**Materials and Methods:**

We successfully established two PeCa cell lines from fresh tumor tissues from 21 cases. One cell line named Penl1 was isolated from a lymph node metastasis (LNM) of penile squamous cell carcinoma (PeSCC), usual type and comprehensively characterized here. Our in-depth characterization analysis of the Penl1 cell line included morphology, tumorigenicity, genetic characteristics, protein expression, biology, and chemosensitivity. Penl1 was authenticated by single tandem repeat (STR) DNA typing.

**Results:**

Comparative histomorphology, genetic characteristics, and protein expression patterns revealed essential similarities between the cell line and its corresponding LNM. In-depth characterization analysis of Penl1 cell line revealed tumorigenicity in immunodeficient mice, negative human papilloma virus (HPV) and mycoplasma infection, TP53 mutations and sensitivity to cisplatin and epirubicin. STR DNA typing did not match any cell lines within three international cell banks. The limitation of this study is that one patient cannot represent the complete heterogeneity of PeCa, especially primary tumor.

**Conclusions:**

We established and characterized an HPV-negative and moderately differentiated PeCa cell model with a TP53 missense mutation from a PeSCC, usual type patient. A preliminarily study of carcinogenesis and chemosensitivity suggests that this cell model carries a tumor suppressor gene mutation and is sensitive to chemotherapy drugs.

## INTRODUCTION

Penile squamous cell carcinoma (PeSCC) is responsible for the majority of penile cancer (PeCa), and is a rare urological cancer in developed countries, representing less than 1% of male cancers in the United States [1]. However, PeSCC is not as rare in developing countries including South America, Asia and Africa [2]. Risk factors associated with it include poor hygiene, phimosis, lack of circumcision, tobacco use, and human papillomavirus (HPV) infection [3, 4].

Given that HPV has been recognized as a carcinogenic factor for cervical cancer since the 1970s [5], the carcinogenic role of HPV in PeCa is supported only by retrospective clinical study [6]. No studies of the role of HPV in penile carcinogenesis based on cell models are currently available.

Due to the lack of suitable cell models, progress in the basic study in PeCa is difficult, and the development of new therapies is at a standstill. *In vitro* cell models are indispensable for a deep understanding of carcinogenesis and metastasis; however only a few cell lines isolated from primary lesions or metastasis are available for study [7–10]. In 1989, Takuji et al established a PeSCC cell line, KU-8, from a lymph node metastasis (LNM) and characterized its epithelial origin [9]. The secretion of SCC-related antigen (SCC-RA) and its effect on cell growth with epidermal growth factor (EGF) was studied in this cell line. In 2012, Naumann et al. established a pair of cell lines from primary tumor and its corresponding LNM. They investigated the role of chemokines, chemokine receptors and podoplanin (PDPN) in this pair of cell lines [10]. These two reports provided a better understanding of tumor growth and metastasis in PeCa. In 2016, cell cultures and xenografts derived from a human verrucous penile carcinoma were established and characterization [11]. While as we know, verrucous carcinoma account for only 7 % of PeSCC and a well differentiated SCC type with no metastasis [12]. However, there have been no advances in the understanding of its carcinogenesis and therapeutic study, and there are no commercially available cell lines for LNM in PeCa.

In PeCa, the most important prognostic factor is the presence and the extent of lymphatic metastases, rather than the T stage of primary disease [13]. There are many questions with respect to the diagnosis and treatment of regional lymph nodes. Most of patients with pTis, pTaG1-2, or pT1G1 disease do not benefit from radical inguinal lymph node dissection (ILND) and may suffer complications [3]. Adjuvant chemotherapy is required for advanced metastatic disease, but first line cisplatin (DDP)-based chemotherapy achieved response rates of only 30 to 35% and short overall survival [14]. Diagnostic and therapeutic advances for PeCa associated LNM have not been achieved. Additional studies of chemotherapy regimens and targeted therapy are needed [15].

As a part of this process, we established a novel PeSCC cell line, designated Penl1, from a LNM of a Chinese PeCa patient. Subsequently, we systematically investigated the cellular morphology, tumorigenicity, DNA typing, HPV or mycoplasma infection, TP53 mutation, protein expression, biology and chemosensitivity of this cell line.

## RESULTS

### Establishment and characterization of a new PeSCC cell line Penl1

Fresh tumor tissues from 21 PeCa patients were cultured in our laboratory (Supplementary Table S1). We established two cell lines, Penl1 and Penl2, through mechanical disaggregation and high-glucose Dulbecco's Minimum Essential Medium (DMEM) supplemented with 10% fetal bovine serum (FBS) as culture medium, with a success rate of 9.52% (2/21). Penl1 was stably passaged in the first 3 months and comprehensively characterized here.

**Figure 1:**
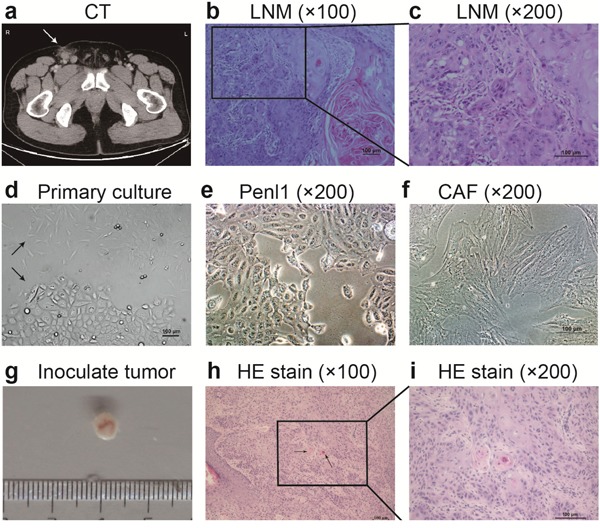
Morphology of LNM, Penl1 cells and their inoculated tumors in SCID mice **a.** Computed tomography (CT) of the patient revealed a 17-mm enlarged lymph node in the right groin area, which was considered to be metastasis. **b**, **c.** HE staining revealed the resected-enlarged lymph node that was confirmed to be penile moderately differentiated squamous cell carcinoma, usual type. **d.** Representative area depicting epithelial-like and fibroblast cells grown in the same flask at passage 0. **e.** Epithelial cells that were isolated as Penl1 grew as monolayer in a cobblestone pattern with distinct epithelial appearance without fibroblasts. **f.** Fibroblast cells isolated as CAFs were fusiform or star-shaped. **g-i.** Inoculated tumor grafts were approximately 5 μm, and keratin pearls (arrow) were observed in HE staining, confirming moderately differentiated squamous cell carcinoma, usual type.

The Penl1 cell line was derived from a LNM specimen from a 41-year-old male with PeCa pathologically diagnosed with PeSCC at Sun Yat-sen University Cancer Center (SYSUCC) (Figure 1a, 1b, 1c). In the first three days, populations of epithelial-like cells and fibroblast cells were grown directly from the tumor tissue fragments (Figure 1d) Epithelial cells were successfully purified at passage 6 and cultured for more than 14 months *in vitro*. Cancer associated fibroblasts (CAFs) cultured for limited passages and stopped growing at approximately passage 14.

The well-attached Penl1 cells grew as a monolayer in a cobblestone pattern with a distinct epithelial appearance and exhibited typical characteristics of malignancy (Figure 1e). CAFs were fusiform or star-shaped cells with multi-branches (Figure 1f). Furthermore, ultrastructural analysis of Penl1 cells revealed desmosomes in intercellular connections and tonofilaments in the cytoplasm (Supplementary Figure S1). Immunocytochemistry indicated that Penl1 cells were pan-cytokeratin (CK) positive and Vimentin negative, whereas CAFs were pan-CK negative and Vimentin positive (Supplementary Figure S2).

Penl1 cells were tumorigenic in severe combined immunodeficiency (SCID) mice. Subcutaneous tumors developed in two out of three mice and were confirmed to be PeSCC by hematoxylin and eosin (HE) staining (Figure 1g). The inoculated tumor exhibited the histological characteristic of moderately differentiated PeSCC, usual type, with few keratin pearls (Figure 1h, 1i). Metastases in lymph nodes and visceral organs were not detected in mice.

**Figure 2:**
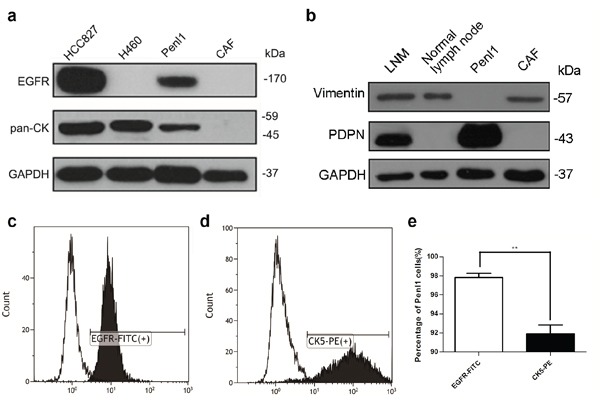
Protein expression and purity of Penl1 cells **a**, **b.** Penl1 expressed pan-CK and EGFR compared with HCC827 and H460 cells lysates. Western blot of original LNM, normal lymph nodes, Penl1 and CAF cells revealed the expression of Vimentin and PDPN. **c-e.** Fluorescence-activated cell sorting (FACS) confirmed that 98.0% of Penl1 cells express EGFR, and these levels were higher than CK5 expression (**, p = 0.0095).

Western blot analysis revealed the protein expression of epidermal growth factor receptor (EGFR) and pan-CK in Penl1 cells compared with HCC827 and H460 (lung adenocarcinoma cell lines; HCC827 was used as a positive control, and H460 was used as a negative control for EGFR-overexpression [16]) (Figure 2a). Vimentin and PDPN expression was also evaluated in original metastatic and normal lymph nodes, Penl1, CAFs and HeLa cells (Figure 2b). Penl1 cells were pan-CK positive and Vimentin negative, which confirmed their epithelial origin. The purity of Penl1 cells was 98.0% as determined by flow cytometry using EGFR-FITC staining; the percentage of CK5 positive cells was 92.7% (**p = 0.0095) (Figure 2c-2e).

**Figure 3:**
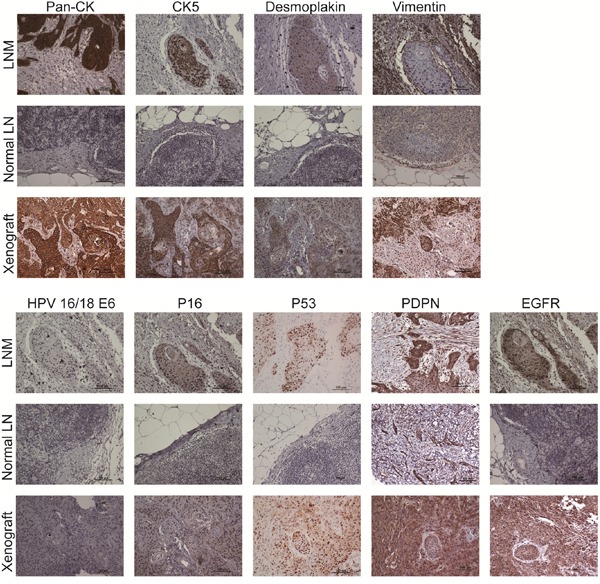
Immunophenotype of indicated cellular biomarkers and HPV subtype markers among LNM, normal lymph nodes and xenografted tumors The epithelial markers, pan-CK, CK5 and desmoplakin, were positive in LNM and xenografted tumors but negative in normal lymph nodes. Vimentin, a mesenchymal marker, was positive in a portion of LNM, normal lymph nodes and xenografted tumors. HPV 16/18 E6 was negative in all sections. Weak, diffuse and focally scattered positive staining for p16 was observed in LNM and xenografted tumors. Positive p53 nuclear expression was noted in LNM and xenografted tumors. Membrane expression of PDPN and EGFR was also positive in LNM and xenografted tumors.

The staining patterns of pan-CK, CK5, desmoplakin, HPV 16/18 E6, p16, EGFR, PDPN, and p53 were consistent with LNM and inoculated tumors (Figure 3). Inoculated tumor tissue section were partially stained for Vimentin. All immunophenotype markers were negative in normal lymph nodes except for Vimentin.

The STR profiles of Penl1, its corresponding tumor and normal lymph nodes (Table 1) are generally consistent and did not match any cell lines within the American Type Culture Collection (ATCC), Deutsche Sammlung von Mikroorganismen und Zellkulturen GmbH (DSMZ) or the Japanese Collection of Research Bioresources (JRCB) database (https://www.dsmz.de/).

**Table 1:** STR profile of Penl1 cell line, compared to original LNM and normal lymph node

Loci	LNM	Normal lymph node	Penl1
Amelogenin	X,Y	X,Y	X,Y
CSF1PO	12	12	12
D13S317	9,12	9,12	9,12
D16S539	11,12	11,12	11,12
D5S818	11,13	11,13	13
D7S820	11	11	11
THO1	7,9	7,9	7,9
TPOX	8,11	8,11	8,11
vWA	18	18	18

### HPV-induced carcinogenesis in Penl1 cell line

Penl1 cells did not exhibit contamination with mycoplasma and HPV DNA based on nest-polymerase chain reaction (nest-PCR) and were HPV-negative (Supplementary Figure S3). These results were consistent with corresponding tumors, normal lymph nodes and CAFs.

**Figure 4:**
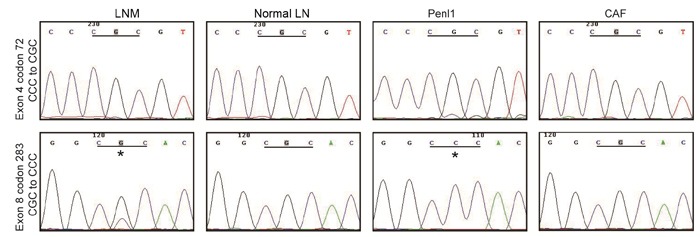
Two TP53 mutations in Penl1 cells Comparative TP53 sequencing results from LNM, normal lymph nodes, Penl1 and CAF cells revealed a SNP in exon 4 (P72R, upper panel) and a missense mutation in exon 8 (R283P, lower panel) in Penl1 cells. The “*” indicates the mutation site of exon 8 in the Penl1 cell line and LNM.

Sequencing of TP53 exon amplicons revealed two TP53 mutations in the Penl1 cell line based on the TP53 sequence (Transcript ID: ENST00000269305) (Figure 4). Codon 72 of exon 4 had a CCC/CGC (Pro/Arg) single nucleotide polymorphism (SNP), which was consistent with online data (asia.ensembl.org). Codon 283 of exon 8 had a CGC (Arg) to CCC (Pro) missense mutation, which was a deleterious and non-functional mutation according to the International Agency for Research on Cancer (IARC) TP53 Database (p53.iarc.fr). Other exons had no mutations. The codon 283 mutation R283P existed only in LNM and tumor cell lines.

### Biology of the Penl1 cell line

Penl1 cells were polyploid compared with HeLa and diploid lymphocytes (Supplementary Figure S3a-S3d). However, Penl1 cells exhibited an increased proliferation index (*p = 0.0376) compared with HeLa cells.

**Figure 5:**
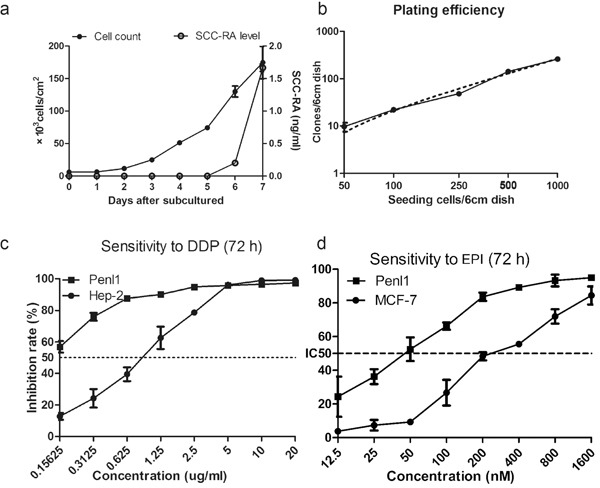
Biology of Penl1 cells **a.** Growth rate curves determined by counting revealed that the population doubling time was 27 hours. SCC-RA levels in culture medium can be detected at day 5. SCC-RA levels increased continuously up to 1.7 ng/ml at day 7. **b.** The plating efficiency curve of Penl1 cells revealed that their plating efficiency was 27.12% (95% confidence interval: 24.39 to 29.86%). **c**, **d.** IR of Penl1 exposed to DDP and EPI, after being cultured in different concentrations of DDP and EPI for 72 hours.

The population doubling time of Penl1 cells was approximately 27 hours at a seeding density of 6 × 10^3^ cells/cm^2^ (Figure 5a). Penl1 cells started secreting SCC-RA at day 5 when the density was 7.4 × 10^4^/cm^2^. The evaluated level of SCC-RA continuously increased up to 1.7 ng/ml, which was beyond clinical cutoffs (1.5 ng/ml). The plating efficiency of Penl1 was 27.12% (Figure 5b) at a seeing density of 50 to 1000 cells/flask.

### Therapeutic agents of Penl1 cell line

The half maximal inhibitory concentrations (IC50) of DDP and epirubicin (EPI) of Penl1 were 0.82 μg/ml and 44.46 nM for 72 hours of treatment, respectively (Figure 5c, 5d). Both drugs were tested and compared with the sensitive cell lines, Hep-2 and MCF-7 [17, 18].

## DISCUSSION

We successfully established a moderately-differentiated PeSCC cell line, Penl1, from a 41-year-old Chinese male (Stage IIIb, T1bN2M0G3). The histological type of the patient was SCC usual type, the most common subtype in PeCa. This cell line was characterized by protein and nucleoid comparison with the original LNM. Our analysis revealed that this cell line was tumorigenic and grows quickly *in vitro*.

Oncogenic viruses, such as HPV, Epstein-Barr virus (EBV) and hepatitis B virus (HBV), are believed to play an important role in tumorigenesis. Cervical cancer is the first cancer known to be associated with HPV due to high prevalence of HPV (greater than 95%) in 1972 [5]. HPV-E6 and HPV-E7 were demonstrated to play a key role in HPV-associated carcinogenesis [19], both are essential for the maintenance of its malignant phenotype [20].

PCR is the standard assay to identify HPV DNA as an initial assessment of HPV-induced carcinogenesis [21]. The inactivation of p53 and Rb by HPV-E6 and HPV-E7 cause negative feedback on p16 and can lead to the accumulation of p16 [22]. Immunoreactivity for p16 is used to distinguish HPV-induced cancers from non-HPV-induced cancers [23]. A correct interpretation of p16 staining is clearly defined as continuous strong nuclear and cytoplasmic staining [24]. A combination of PCR analysis and immunoreactivity for p16 was used to identify HPV-induced cancers in our study. Here, the Penl1 cell line and original tumor were negative for HPV DNA. Both the original LNM and the inoculated tumor exhibited no HPV E6 staining and continuous weak cytoplasmic p16 staining, interpretation of negative p16 staining. Thus, we conclude that Penl1 is an HPV-negative cell line derived from an HPV-negative cancer. The HPV prevalence in PeCa is highly variable in different detection methods, histological subtypes and geographic variation, similar to esophageal SCC [25–27]. The most recent research published on *European Urology* on HPV in PeCa showed that about a third to a fourth of penile cancers were related to HPV when considering HPV DNA detection alone or adding an HPV activity marker, respectively [28]. The role of HPV may be important in SCC warty-baslaoid subtype with 75.3% HPV prevalence, but only 14.3% in non-warty-baslaoid subtype, similar to that of Asia population [27]. Here SCC subtype of Penl1 is usual type and this cell line could be representative in HPV-negative PeCa cell model.

In HPV-negative carcinogenesis, gene alteration plays an important role in PeCa carcinogenesis. TP53 is a tumor suppressor gene, and mutations are noted in approximately 50% of human cancers. In PeSCC, the rate of TP53 mutation is 38% (9/24) regardless of histologic type (http://cancer.sanger.ac.uk/cosmic/). Most studies of TP 53 mutations focus on exons 5 to 8, and we also identified a missense mutation, R283P, in exon 8 in the Penl1 cell line. Codon 283 is located in the sequence-specific DNA-binding region (codons 102-292), and the mutation is a deleterious and non-functional mutation. This mutation can be detected only in the original tumor and the Penl1 cell line. Immunohistochemistry revealed p53 overexpression, which is consistent with mutant status and may result in the loss of DNA binding and play a pathogenic role in this cell line. In addition, we identified a SNP on exon 4 of Penl1 cells, which is frequently associated with human cancers and is considered non-pathogenic in PeCa [29, 30]. Tumor suppressor gene alteration is an important carcinogenesis factor in PeSCC. Recent studies have demonstrated that alterations of tumor suppressor genes can be identified only in a low percentage of cervical cancers in the cosmic database. The rate of TP53 mutation is only 5% in cervical cancer, much lower than any other cancers. It appears tumor suppressor gene alterations are quite rare in HPV-induced carcinogenesis but it is not as rare in HPV-negative carcinogenesis.

Positive expression of TP53 is correlates with TP53 mutations and is also a good prognostic marker in PeSCC [31, 32]. Combination of p53 and p16 expression were studied in PeCa, one research divided PeCa into two major pathways of carcinogenesis: p53 expression with p16 negativity identifies HPV-negative cancers and p16 overexpression identities HPV-HR-induced cancer [6]. This research enable us to pay attention to etiologic classification and the classification may one day allows optimal therapy decisions in PeCa. Due to our incomplete understanding of the role and outcome caused by mutant TP53, TP53 mutation-related carcinogenesis, HPV and other gene alterations in PeSCC require further research.

Chronic inflammation may be an important process during the carcinogenesis of PeCa because it shares the activation of COX-2, PGE2 and EGFR with most of the other SCC tumor phenotypes [33, 34]. Our HPV-negative cell line Penl1 may enable us to better understand the role of chronic inflammation and tumor suppressor gene alteration in carcinogenesis. This cell line would allow unique opportunities to enhance the understanding of and identify effective PeCa treatments.

In PeSCC, advanced lymph node involvement is increasingly treated with multimodality therapy incorporating chemotherapy and/or radiation. Chemotherapy is used as preoperative and postoperative adjuvant therapy with a low clinical response rate of 30%, ranging from 20 to 70% [14]. DDP is widely used in cancer chemotherapy and is the most commonly used chemotherapy agent in advanced PeSCC. We investigated the therapeutic sensitivity of Penl1 to DDP and EPI. The therapeutic effect of these drugs was confirmed with our cell line *in vitro*. Our results suggest that EPI, a widely used therapy in breast cancer that has rarely been reported in PeCa, may be an effective chemotherapy agent for PeCa treatment. Further verification by clinical study of EPI treatment and combination of other biological agents for PeCa is warranted.

Two membrane proteins, EGFR and PDPN, were detected in Penl1 and may be used as targets for targeted therapies. EGFR-inhibiting monoclonal antibodies are used as a salvage strategy in PeCa. A retrospective study has demonstrated that patients with advanced PeCa expressing EGFR that received EGFR-targeted therapies and had a favorable overall survival of 9.8 months utilizing conventional chemotherapy [35]. EGFR monoclonal antibody in combination with chemotherapy and/or radiation warrants further study. Given that PDPN is a important membrane protein associated with metastasis and expressed in tumor cells [36, 37], treatment targeting PDPN is an attractive therapeutic strategy for the treatment of LNM.

### Conclusion

In summary, this moderately-differentiated and HPV negative PeCa cell line with a TP53 mutation from lymph node metastasis was tumorigenic and maintained the histological and molecular features of the original tumor when grown *in vitro*. The genetic features of this cell line implied that a tumor suppressor gene mutation is an important carcinogenesis factor in PeSCC. In addition, this cell line is sensitive to chemotherapy, and the therapies targeting EGFR and PDPN are promising.

## MATERIALS AND METHODS

### Specimens and ethic statement

Fresh tumor tissues from 21 patients in SYSUCC diagnosed with PeCa were cultured in our laboratory from 2014-4 to 2015-4 (Supplementary Table S1). During the surgery, a portion of the resected specimen and matched normal tissue were collected. Histology of the resected tumor and normal tissue was confirmed by pathology. All the patients provided ethically approved informed consent for the scientific research use of the specimens prior to surgery.

### Cell line establishment and culture

Multiple disaggregation approaches and different culture mediums were tested (Supplement). Mechanical disaggregation and DMEM supplemented with 10% fetal bovine serum (FBS) was applied to the primary culture of all the specimens. The specimens were immersed in serum-free, high glucose Dulbecco's modified Eagle's medium (DMEM) medium containing 200 units/ml penicillin and 200 μg/ml streptomycin immediately after resection. After quick transit to the laboratory, the specimens were rinsed 3 times with phosphate buffered saline (PBS) and cut into fragments 1 to 3 mm in diameter. The samples were evenly placed in 25-cm^2^ tissue flasks with a small amount of DMEM supplemented with 10% FBS and placed in a 37°C water jacketed incubator with a humidified 5% CO_2_ atmosphere. When the fragments were attached to the flasks 4 hours later, the flasks were turned over, and approximately 5 ml of medium was added.

Adherent cells expanded from the fragments the next day. The medium was carefully refreshed to remove the cells in suspension, such as lymphocytes and erythrocytes, in the first 3 days. To remove fibroblast-like cells, cells were digested with 0.25% trypsin/ethylenediaminetetraacetic acid (EDTA) until the epithelial-like cells remained adherent to flasks as reported [38]. The digested cell suspension was added to three 25-cm^2^ tissue culture flasks continuously at half-hour intervals, and epithelial cells were purified in the last flask. At passage 6, we selected an epithelial cell colony. We passaged the colony and named it Penl1. The fibroblast-like cells were also isolated and named CAF. Cell lines were passaged at 70 to 90% confluence and stored in liquid nitrogen for further usage. Penl1 cells at passage 21 to 25 were used for later characterization.

### Morphological examination

Cultured Penl1 cells were photographed with a phase-contrast microscope (OLYMPUS IX71) weekly. Transmission electron microscopy samples wereprocessed as previously reported [39].

### Tumorigenicity in mice

To confirm the malignant potential of the cell line, 2.5 × 10^6^ Penl1 cells suspended in 100 μl PBS were injected subcutaneously into the right groin of four-week-old female severe combined immunodeficiency (SCID) beige mice (Vital River, a Charles River company, China). Tumor bearing mice were sacrificed after 6 weeks and tumors were fixed with 10% formalin and sectioned for subsequent HE and immunochemical staining.

### Cell authentication and DNA content

Genomic DNA was isolated from specimens and cells using an AllPrep^®^DNA/RNA/Protein Mini (Qiagen, Germany). The DNA of Penl1 was amplified using the domestic Goldeneye20A Kit (Peoplespot Inc, China). The cell line samples were processed with the ABI3730xl Genetic Analyzer, analyzed with GeneMapper 4.0 software and then compared with the STR profile from the ATCC, DSMZ or JRCB databases for reference matching. The Genewiz Inc. DNA content assay, which was processed as previously described using the Cell Cycle and Apoptosis Analysis Kit (Bestbio, China) [40].

### PCR and TP53 sequencing

Normal and metastatic lymph nodes as well as Penl1 and CAF cells were screened for mycoplasma infection using the PCR Mycoplasma Test Kit (HuaAn Biotech, China). DNA from LNM, normal lymph nodes, Penl1 and CAF cells was also amplified and screened for HPV content through nested PCR using the GoTaq® Green Master Mix (Promega, USA) with consensus primers MY09/MY11 and GP5+/GP6+ [41]. Hep-2 and HeLa cells were analyzed in parallel as positive controls. All PCR products were electrophoretically separated on agarose gels, visualized using Tiangen GeneGreen nucleic acid dye (Tiangen, China), and imaged with the Gel Doc XRt System (BioRad, USA).

Genomic DNA of Penl1 was subjected to PCR amplification of fragments (containing exons 1–11 of TP53) suitable for sequencing [39]. PCR products were gel purified and sequenced from both ends in duplicate by Life technology.

### Protein assay

For Western blots, approximately 20 μg of protein samples from lysates of Penl1 cells and CAF cells were analyzed by sodium dodecyl sulfate–polyacrylamide gel electrophoresis (SDS–PAGE) (Beyotime, China), electrotransferred to polyvinylidene fluoride membranes (Millipore, USA) and probed overnight with primary antibodies against GAPDH, pan-CK, EGFR, p16, PDPN and Vimentin (Santa Cruz, USA). Secondary antibodies (goat anti-mouse/rabbit IgG (H+L), Santa Cruz, USA) were then applied. Signal detection was performed using BeyoECL Plus (Beyotime, China) according to the manufacturer's instructions.

Penl1 cells were harvested, washed and fixed using the Fixation/Permeabilization Solution Kit (BD, Cat. No. 554715, USA) for flow cytometry. After fixation and permeabilization, cells were incubated in wash buffer containing CK5 (Santa Cruz, USA) for 45 min at 4°C, and normal anti-mouse IgG1 was used as an isotype control. The cells were then washed with wash buffer and incubated with anti-mouse IgG1-PE for 30 min at 4°C. Cells were also incubated with anti-EGFR-FITC for 30 min at 4°C (Miltenyi Biotec, Germany); normal rabbit IgG-FITC was used as an isotype control. The cells were analyzed using a Gallios flow cytometer (Beckman Coulter, USA).

For immunocytochemistry preparation and staining, Penl1 cells and CAFs were grown for 12 hours on glass coverslips in six-well plates. The cells were fixed with 4% paraformaldehyde (PFA) for 10 min, permeabilized with 0.3% Triton X-100 and incubated in 5% bovine serum albumin (BSA) at room temperature for 60 min to block nonspecific interactions. To validate the epithelial and mesenchymal origin of the cells, Penl1 and CAFs were stained with pan-CK (Santa Cruz, USA), Vimentin (Santa Cruz, USA), as described in the following immunochemical staining section. The results were photographed with a Nikon Eclipse 80i microscope microsystem.

Blocks of LNM, normal lymph nodes from the patient and inoculated tumors from SCID mice were fixed in 10% formalin for 24 hours, embedded in paraffin, and then cut into 3-μm tissue sections. The sections were then affixed to slides, deparaffinized, rehydrated through graded alcohols, heated in a microwave oven on high power and medium power for 10 min in 10 mM EDTA (pH 9.0) for antigen retrieval, incubated in 3% H_2_O_2_ for 10 min to inactivate endogenous peroxidase, and incubated in 5% BSA for 40 min. After blocking, the tissue sections were incubated with primary antibodies overnight at 4°C, followed by the addition of secondary antibodies and incubation in the dark for 1 hour at 37°C. For tissue slides, a SignalStain® 3,3 N-Diaminobenzidine (DAB) Tertrahydrochloride Substrate Kit was used after incubation with secondary antibodies to visualize target proteins, and Van-Hematoxylin hematoxylin stain (Harris) was used for counterstaining. Observation and image acquisition were performed using a NIKON ECLIPSE 80i advanced research microscope. Primary antibodies against pan-CK, Vimentin, CK 5, desmoplakin, HPV16 E6/18 E6, p16 (Santa Cruz, USA), p53 rabbit mAb (Cell Signaling Technology, USA), and EGFR (Santa Cruz, USA) were used. The secondary antibodies included peroxidase-conjugated goat anti-mouse (Boster, China).

### Growth and plating efficiency assay

Penl1 cells were seeded in 25-cm^2^ flasks at a density of 6 × 10^3^ cells/cm^2^ in 3 replicates. At day 1, 2, 3, 4, 5, 6, and 7 after seeding, cells in dishes were harvested and counted in a hemocytometer chamber. The media was refreshed every 24 hours, and the older culture media was collected for the detection of SCC-RA using clinical methods (immunoradiometric assay). SCC-RA was detected in the culture medium at 24 hours.

Cell plating efficiency represents the cell proliferation and survival of cells at low density (20-50 cells/cm^2^). Briefly, 50, 100, 250, 500, and 1000 cells were seeded in 6-cm cell culture dishes in triplicate, and 10000 cells were seeded in a dish of the same size as a control to ensure colony formation. Colonies could be observed after approximately 1 week. Colonies were dyed with crystal violet and photographed. Colonies of more than 16 cells were calculated.

### Drug sensitivity assay

Penl1 cells were seeded at 5 × 10^3^ cells/well in 96-well plates and cultured in culture medium containing DDP (Pharmaceutical Factory, China) in a concentration gradient (0, 0.5, 1, 3, 5, 7, 9, 11, and 13 μg/ml) and EPI (Pfizer, USA) in a concentration gradient (6.25, 12.5, 25, 50, 100, 200, 400, 800, and 1600 nM). Each concentration was repeated in 3 wells. An additional 6 wells, containing culture medium only, served as a control. After culture for 72 hours, the cells were assessed by incubating with CCK-8 for 2.5 hours. The inhibition rate (IR) was evaluated using the formula IR = 100% - survival rate (SR), and the SR was measured using the formula SR = (mean absorbance of the test wells/mean absorbance of the control wells) × 100%.

### Statistical analysis

Data were presented as the mean ± standard deviation (SD) of at least three independent experiments. The Student's t test was used to examine differences using GraphPad Prism software (version 5.00 for Windows; GraphPad Software); p-values < 0.05 were considered significant.

## SUPPLEMENTARY MATERIAL and Figures




