# Supplementary material for: Establishment and characterization of a penile cancer cell line, penl1, with a deleterious TP53 mutation as a paradigm of HPV-negative penile carcinogenesis
Source: Oncotarget. 2016 Jun 16;7(32):51687–98. doi: 10.18632/oncotarget.10098 (PMC5239507; doi:10.18632/oncotarget.10098)
Supplement: Supplementary file 1 [file oncotarget-07-51687-s001.pdf]

Establishment and characterization of a penile cancer cell line, penl1, with a deleterious TP53 mutation as a paradigm of HPV-negative penile carcinogenesis

SUPPLEMENTARY MATERIAL

Disaggregation approaches and medium selection

Multiple approaches, including mechanical disaggregation and enzymatic dissociation with trypsin-EDTA or collagenase and different mediums (DMEM, M199, RPMI-1640, or keratinocyte/serum-free medium) were performed to optimize culture condition in pre-test. Different FBS concentration, ranging from 2-20%, were used in the culture medium and finally 10% FBS was the best concentration for Penl1.

Patient of Penl1 cell line-

The patient was diagnosed with PeSCC in September 2013 because of a non-healing ulcer on penis after circumcision, had partial penectomy and right inguinal lymph node excisional biopsy in other hospital. He complained with fixed right inguinal lymph nodes mass 6 months later. The pathology of specimens was consulted as moderately to poorly differentiated PeSCC, negative margin and no right inguinal lymph nodes involvement by pathology. Recent pelvic CT found a 17 mm enhanced lymph node on the right groin area with unclear boundary and considered as metastasis (Figure. 1a). No local recurrence was found when palpable lymph nodes emerged after penectomy. Of note, the subject had a 38-year history of childhood phimosis and a 30-year history of alcohol use. Preoperative and postoperative SCC-RA levels of the patient were normal. He had bilateral inguinal lymphadenectomy with preservation of the fascia lata [22] at SYSUCC in June 2014 and specimen of the LNM was cultured *in vitro*. As lymphadenectomy confirmed the pN2 with three lymph node metastases showing moderately differentiated PeSCC (Figure. 1b, 1c), after cisplatin-based adjuvant chemotherapy, the patient had prophylactic pelvic lymphadenectomy and pathology was negative. The patient is still alive without recurrence.

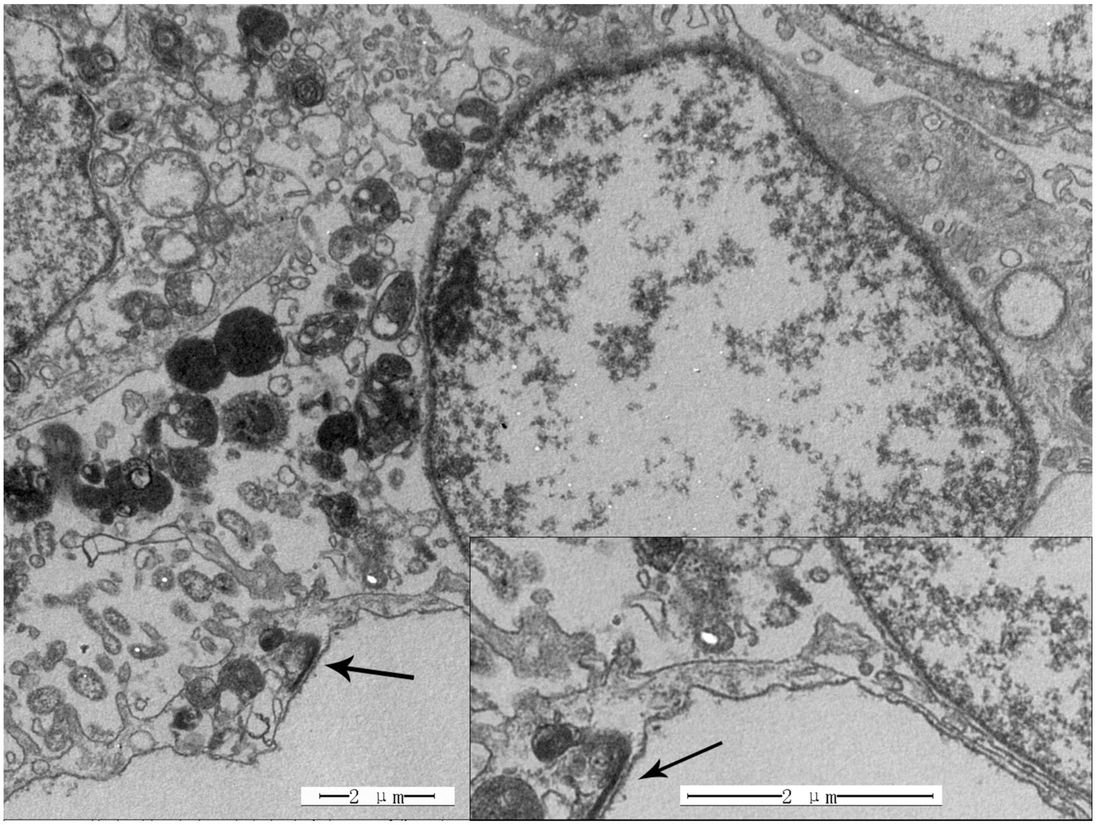

Supplementary Figure S1: Penl1 cells showed apparent tonofilaments in the cytoplasm and intercellular desmosomes (black arrow).

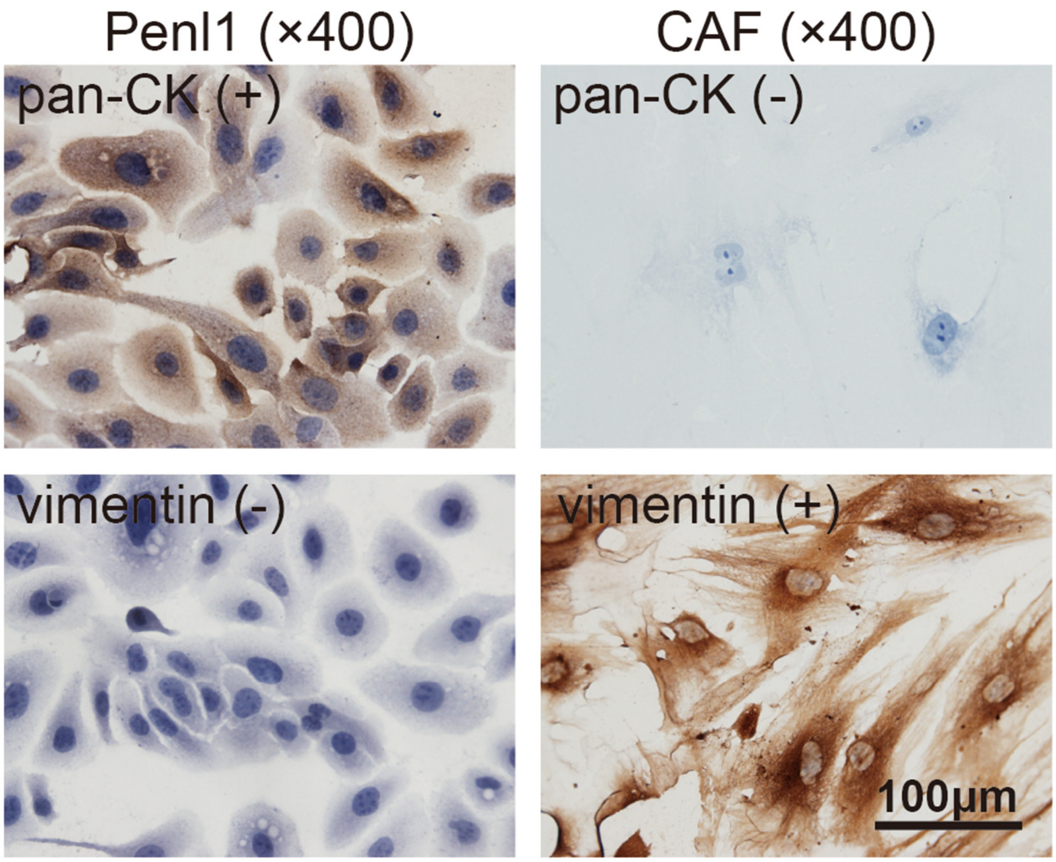

Supplementary Figure S2: Penl1 cells were pan-CK-positive and vimentin-negative (upper panel), while CAF cells were pan-CK-negative and vimentin-positive (lower panel).

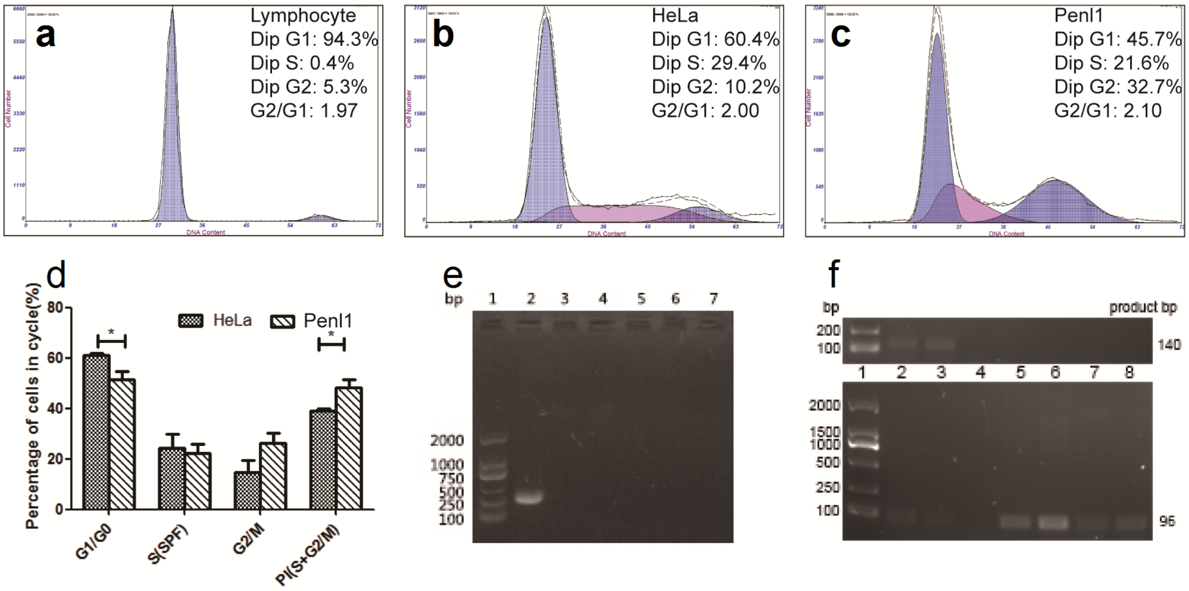

Supplementary Figure S3: DNA content and HPV status of Penl1 cells. a-c. DNA content of Penl1 cells determined by flow cytometry were polyploidy compared with the diploid lymphocyte (a) and HeLa (b). Statistical analysis **d.** showed that Penl1 cells had a higher PI (*P =0.0376) compared with HeLa cells. **e.** PCR detection result showed absence of mycoplasma contamination of (4) origin LNM, (5) normal lymph node, (6) Penl1and (7) CAF cells compared with (2) positive control (290bp) and (3) water; (1) DL2000 DNA marker. **f.** HPV detection using GAPDH primers as a quality control (lower panel), Two HPV consensus primers MY09/MY11 and GP5+/GP6+ were used in the nested PCR and result showed that (5) origin LNM, (6) normal lymph node, (7) Penl1and (8) CAF cells were HPV-negative compared with (2) Hep-2 and (3) HeLa cells and (4) water; (1) DL2000 DNA marker.

Supplementary Table S1: Characteristics of primary culture from 21 patients with penile cancer

See Supplementary File 1
