# Supplementary material for: Establishment and characterization of a penile cancer cell line, penl1, with a deleterious TP53 mutation as a paradigm of HPV-negative penile carcinogenesis
Source: Oncotarget. 2016 Jun 16;7(32):51687–98. doi: 10.18632/oncotarget.10098 (PMC5239507; doi:10.18632/oncotarget.10098)
Supplement: Supplementary file 2 [file oncotarget-07-51687-s002.docx]

**Supplementary Table 1 Characteristics of primary culture from 21 patients with penile cancer**

| Pt No | Age | TNM | Grade | Histological type | SCCAg/ng/ml | HPV status | Surgery | Specimen | Time start | Result |
| --- | --- | --- | --- | --- | --- | --- | --- | --- | --- | --- |
| 1 | 35 | pT1N3M0 | 3 | SCC, usual type | 9.6 | - | BILND and BPLND after circumsicsion and LNB | LNM | Apr-14 | Abandoned 2.5 m later |
| 2 | 55 | pTxN3M1 | - | SCC, usual type | - | - | BILND after PP and BILND | LNM | Apr-14 | Abandoned 2 m later |
| 3 | 47 | pTaN0M0 | 1 | SCC, usual type | 0.4 | - | PP and BILND after glansectomy | PT | May-14 | Abandoned 1 m later |
| 4 | 41 | pT1aN2M0 | 2 | SCC, usual type | 1.1 | - | BILND after PP and LNB | LNM | Jun-14 | Alive, 80st passage |
| 5 | 62 | pT1aN3M0 | 2 | SCC, usual type | 1.8 | - | TP after glansectomy, BILND and BPLND | PT | Jun-14 | - |
| 6 | 50 | pT2N2M0 | 1 | SCC, usual type | 1.3 | - | BILND after TP | LNM | Jun-14 | Alive, 44st passage |
| 7 | 49 | pT1N3M0 | - | SCC, usual type | 4.2 | - | BILND after PP | LNM | Jun-14 | - |
| 8 | 42 | pT4N3M1 | 2 | SCC, usual type | - | - | skin metastasis biospy after TP, BILND and BPLND | metastasis | Aug-14 | - |
| 9 | 42 | pT2N1M0 | 2 | SCC, usual type | 0.5 | - | TP after PP and BILND | PT | Aug-14 | Infection |
| 10 | 72 | pT2N3M0 | 2 | SCC, usual type | 3.1 | - | TP, BLND and BPLND after PP | PT | Aug-14 | - |
|  |  |  |  |  |  | - |  | LNM |  | - |
| 11 | 36 | pT2N1M0 | 1 | SCC, usual type | 4.3 | - | TP and BILND after tumor biospy | PT | Sep-14 | Infection |
| 12 | 58 | pT2N0M0 | 3 | SCC, usual type | 3.2 | - | PP and BILND after tumor biospy | PT | Sep-14 | Abandoned <1 m later |
| 13 | 33 | pT3N2M0 | 1 | SCC, usual type | 0.6 | - | BPLND after PP and BILND | LNM | Sep-14 | - |
| 14 | 59 | pTxN3M0 | 2 | SCC, usual type | 1 | - | BILND and BPLND after PP | LNM | Sep-14 | - |
| 15 | 45 | pT1aN0M0 | 1 | SCC, usual type | 4.6 | negative | PP and BILND after tumor biospy | PT | Oct-14 | - |
| 16 | 88 | pT2N0M0 | 2 | SCC, usual type | 15.6 | - | PP | PT | Nov-14 | - |
| 17 | 49 | pT2N0M0 | 2 | SCC, usual type | 0.4 | - | PP after circumcision | PT | Jan-15 | - |
| 18 | 41 | pT2N0M0 | 1 | SCC, usual type | 0.6 | - | PP after tumor biospy | PT | Feb-15 | - |
| 19 | 61 | pT2N0M0 | 2 | SCC, usual type | 1.2 | negative | PP and BILND after biospy | PT | Mar-15 | Abandoned <1 m later |
| 20 | 62 | pT2N0M0 | 1 | SCC, papillary NOS | 27.9 | negative | PP and BILND | PT | Mar-15 | - |
| 21 | 38 | pT1aN0M0 | 1 | SCC, usual type | 0.8 | negative | PP and BILND after biospy | PT | Apr-15 | - |

**#: 2010 TNM clinical and pathological classification of penile cancer.**

**SCC: squamous cell carcinoma; NOS: not other specified; BILND: bilateral inguinal lymphadenectomy; BPLND: bilateral pelvic lymphadenectomy; PP: partial penectomy; TP: total penectomy; LNB: lymph node biopsy; LNM lymph node metastasis; PT: primary tumor; “-” means the data is not available.**
